# Determination of the effective dose of dexmedetomidine to achieve loss of consciousness during anesthesia induction

**DOI:** 10.3389/fmed.2023.1158085

**Published:** 2023-04-20

**Authors:** Bing Mu, Wenjie Xu, Hongyi Li, Zizheng Suo, Xiaoxiao Wang, Yuxiang Zheng, Yi Tian, Bowen Zhang, Jie Yu, Naiyuan Tian, Nan Lin, Dan Zhao, Zhaoxu Zheng, Hui Zheng, Cheng Ni

**Affiliations:** ^1^Department of Anesthesiology, National Cancer Center/National Clinical Research Center for Cancer/Cancer Hospital, Chinese Academy of Medical Sciences and Peking Union Medical College, Beijing, China; ^2^Clinical Epidemiology Research Center, Peking University Third Hospital, Beijing, China; ^3^Department of Neurology, Peking Union Medical College Hospital, Peking Union Medical College and Chinese Academy of Medical Sciences, Beijing, China; ^4^Department of Gynecology Oncology, National Cancer Center/National Clinical Research Center for Cancer/Cancer Hospital, Chinese Academy of Medical Sciences and Peking Union Medical College, Beijing, China; ^5^Department of Colorectal Surgery, National Cancer Center/National Clinical Research Center for Cancer/Cancer Hospital, Chinese Academy of Medical Sciences and Peking Union Medical College, Beijing, China

**Keywords:** dexmedetomidine, anesthesia induction, effective dose, electroencephalogram, patient state index

## Abstract

**Background:**

Dexmedetomidine (DEX) is a sedative with greater preservation of cognitive function, reduced respiratory depression, and improved patient arousability. This study was designed to investigate the performance of DEX during anesthesia induction and to establish an effective DEX induction strategy, which could be valuable for multiple clinical conditions.

**Methods:**

Patients undergoing abdominal surgery were involved in this dose-finding trial. Dixon's up-and-down sequential method was employed to determine the effective dose of DEX to achieve the state of “loss of consciousness”, and an effective induction strategy was established with continuous infusion of DEX and remifentanil. The effects of DEX on hemodynamics, respiratory state, EEG, and anesthetic depth were monitored and analyzed.

**Results:**

Through the strategy mentioned, the depth of surgical anesthesia was successfully achieved by DEX-led anesthesia induction. The ED50 and ED95 of the initial infusion rate of DEX were 0.115 and 0.200 μg/kg/min, respectively, and the mean induction time was 18.3 min. The ED50 and ED95 of DEX to achieve the state of “loss of consciousness” were 2.899 (95% CI: 2.703–3.115) and 5.001 (95% CI: 4.544–5.700) μg/kg, respectively. The mean PSI on the loss of consciousness was 42.8 among the patients. During anesthesia induction, the hemodynamics including BP and HR were stable, and the EEG monitor showed decreased α and β powers and increased θ and δ in the frontal and pre-frontal cortices of the brain.

**Conclusion:**

This study indicated that continuous infusion of combined DEX and remifentanil could be an effective strategy for anesthesia induction. The EEG during the induction was similar to the physiological sleep process.

## Introduction

Dexmedetomidine (DEX) is a highly selective α2 adrenergic receptor agonist with sedative, hypnotic, and anxiolytic effects, which are widely used in ICU for sedation and in clinical anesthesia during surgical procedures (1). DEX could alter the patient's state of arousal through its actions on presynaptic α2 receptors on neurons projecting from the locus coeruleus. DEX's binding of α2 receptors hyperpolarizes locus coeruleus neurons and decreases norepinephrine release, which results in loss of inhibitory inputs to the preoptic area of the hypothalamus (2). The preoptic area sends GABAergic and galanergic inhibitory projections to the major arousal centers in the midbrain, pons, and hypothalamus. Hence, loss of the inhibitory inputs from the locus coeruleus results in sedation due to the activation of these inhibitory pathways from the preoptic area to the arousal centers (3, 4). Studies have indicated that DEX-induced deep sedation neurophysiologically approximates non-rapid eye movement (NREM) sleep (5). Anesthesia induction led by DEX may lead to a process that approximates natural sleep-related electroencephalogram (EEG) dynamics and becomes a natural and valuable induction strategy. Furthermore, DEX could also contribute to the prevention of postoperative cognitive dysfunction (POCD) (6).

Induction of general anesthesia is often accompanied by fluctuations in hemodynamics which may result in perioperative organ hypoperfusion that increases the postoperative morbidity and mortality risks (7), especially for patients with tumors whose tolerance to stress is worse. Commonly used anesthetic propofol induces cardiovascular depression with a decrease in blood pressure (BP), and 22.6% of patients undergoing propofol-led anesthetic induction experienced pre-incision severe hypotension (MAP ≤ 55 mmHg) (8). Intravenous DEX induces a biphasic BP response through peripheral vasoconstriction. Modest declines in heart rate (HR) and BP are observed at lower DEX concentrations, but bradycardia and hypertension at higher concentrations (9). Meanwhile, the central inhibition of the sympathetic system by DEX could minimize perioperative patient stress and BP instability and improve hemodynamic control (10). DEX produces sedation and anxiolysis through its inhibitory effect on α2 receptors, and it has minimal effects on respiratory drive (11). Respiratory depression and restlessness are rare during DEX sedation, and DEX has potential benefits for patients with a difficult airway. Michael et al. (12) reported that DEX could complete anesthesia induction in difficult airway patients without a significant oxygen saturation decrease. Furthermore, DEX could affect neuroinflammation in the hippocampus through the glial cell and TLR4 inhibition, thereby preventing neuronal apoptosis and POCD. Thus, DEX has potential benefits for patients with neurodegenerative diseases such as Alzheimer's disease (13, 14). DEX also has minimal impact on evoked potential monitoring and unique advantages for neurosurgical procedures which require neurological examination (15). Based on the above advantages, DEX-led anesthesia induction protocols could be valuable for multiple contexts and patients.

Compared with midazolam, DEX provided similar sedative effects and better analgesic effects (16). A previous study indicated that continuous DEX infusion reduced the propofol requirement to achieve the same anesthetic depth (17), which suggested that DEX has the potential to become a major part of general anesthesia strategy. As a dose-dependent sedative, if enough dose is provided, DEX could produce deep sedation or general anesthesia, but a higher dose of DEX may also lead to an increased risk of bradycardia and hypertension (18). Numerous factors such as hypoalbuminemia, liver dysfunction, reduced cardiac output, and hemodynamic alterations affect metabolic clearance and distribution volume, thereby influencing the DEX dosage (19, 20). Therefore, the doses of DEX in anesthesia induction and the individual variations of DEX doses could both be large and need investigations. In the present study, we sought to clarify the feasibility of DEX for anesthesia induction and tried to establish an induction strategy led by DEX, and the primary aim was to determine whether the depth of anesthesia induction dominated by DEX is sufficient for surgical operation, and further determine the effective dose of DEX to achieve “loss of consciousness (observer's vigilance/sedation assessment, OAA/S score=1)” during induction. The cardiovascular effects of DEX were controlled through infusion rate modification and combination with remifentanil, and the secondary aim was to establish an effective strategy for DEX-led anesthesia induction, to investigate the suitable infusion rate, induction time, hemodynamics, EEG characteristics, and patient state index (PSI) changes during DEX induction.

## Methods

The study was conducted at the Cancer Hospital, Chinese Academy of Medical Sciences from April to July 2022. After we obtained approval from the ethics committee of the National Cancer/Cancer Hospital, Chinese Academy of Medical Sciences, and Peking Union Medical College (Approval No. 21/024-2695), we registered the study at the Chinese Clinical Trial Registry (Registration No. ChiCTR2200055246). A total of 36 patients aged 28–59 years and ASA physical status I or II undergoing abdominal surgery for gastric, colorectal, and gynecologic tumors were recruited after gaining written informed consent. The patients were excluded if one of the following criteria is true: BMI < 18.5 or > 30 kg/m^2^, sinus bradycardia (HR < 50 bpm), severe heart block (third-degree heart block or type II second-degree heart block), cardiac insufficiency (LVEF < 50%), severe valvular heart disease, systemic or important organ infection, severe anemia, severe liver, renal or thyroid dysfunction, neurological disease or having received central nervous system drugs, preoperative cognitive dysfunction (mini-mental state examination, MMSE < 24), long-term use of α adrenergic receptor-blocking drugs, and allergy to the anesthetic drugs used in this study. The age, sex, body weight, height, temperature, levels of ALT, AST, creatinine, urea, Hgb, MMSE, personal history, ASA grade, and comorbidities were collected and recorded on the case report forms before surgery.

All patients had no premedication. Upon arrival at the operating room, monitors including electrocardiography, pulse oximetry, and BP were applied. SedLine electroencephalograph sensors (SedLine Sedation Monitor, Masimo Co., USA) were attached to the forehead area to monitor the patient state Index (PSI) and reflect the anesthetic depth. PSI scores of 50–100 indicate light sedation or being awake, 25–50 indicate surgical anesthesia, and 0–25 indicate an overdose or deep sedation. General anesthesia was performed for all patients. Approximately 6 L/min of oxygen was provided with facemasks for all patients during induction. To improve the induction process and tolerability of tracheal intubation, anesthesia induction was started with 0.3 μg/kg sufentanil, and 30 s later, continuous DEX (Yangtze River Pharmaceutical Group Co., Ltd, Jiangsu, China) and remifentanil infusion (Silugao CP-730 TCI pump, Silugao Med Tech, Beijing, China) were performed. The infusion rate of remifentanil was set at 0.1 μg/kg/min.

Through preliminary experiments, we noticed that starting with a high DEX infusion rate had a larger effect on HR and BP. Thus, the initial DEX infusion rate was set at a relatively low rate for the first 9 min. Then, the infusion rate of DEX was adjusted at two times the initial rate for the second 9 min, and three times the initial rate for the third 9 min, and continued this cycle until achieving the state of “loss of consciousness” (OAA/S scores = 1). Based on the preliminary experiment, this strategy would not significantly affect HR, BP, and spontaneous breath during induction, and there was a ceiling effect for the vasoconstriction effect of DEX (21). Furthermore, we prepared appropriate vasoactive drugs and laid down a criterion to call off the trial for the occurrence of severe and uncorrectable bradycardia. OAA/S score was assessed every 3 min on a scale from 5 (readily responds to name spoken in normal tone) to 1 (no response to mild prodding or shaking, Supplementary Table 1). If OAA/S score > 1, the infusion of DEX and remifentanil was continued. If OAA/S score = 1, the state of “loss of consciousness” was diagnosed. The initial infusion rate and total dose of DEX to achieve the state “loss of consciousness” were recorded and calculated. 3 min interval was relatively short, but if the assessment interval was prolonged, the DEX dose would be larger than the actual situation. HR, BP, and PSI were recorded every 3 min during the induction process.

The initial infusion rate of DEX was determined using Dixon's up-and-down sequential method. Based on the preliminary experiment, the initial infusion rate of the first subject was set at 0.15 μg/kg/min. If the state of “loss of consciousness” was achieved within 18 min, the initial infusion rate of the next subject stepped down by 0.03 μg/kg/min, and if the state of “loss of consciousness” was achieved more than 18 min, the initial infusion rate of the next subject stepped up by 0.03 μg/kg/min. The study and patient recruitment were continued until there were at least six crossover pairs (a successful induction within 18 min followed by a failed induction within 18 min) and 30 subjects were finished.

When OAA/S = 1, adequate anesthesia depth was achieved for intubation and 0.8 mg/kg rocuronium was infused, and the infusion rate of DEX was adjusted to the initial rate of the current subject to maintain anesthesia. Endotracheal intubation was performed, then HR, BP, and PSI were recorded at 1 and 5 min after intubation. During anesthesia induction, 0.5 mg of atropine was infused if bradycardia (HR < 45 bpm) occurred, 100 μg of nitroglycerine was infused if systolic BP (SBP) > 180 mmHg occurred, and jaw thrust or assisted ventilation was performed if SpO_2_ < 95%. These situations were recorded.

Anesthesia maintenance was performed with 0.75–1.5 MAC desflurane, 0.1–0.3 ug/kg/min remifentanil, and 0.3–0.6 mg/kg/h rocuronium. PSI was maintained in the range of 25 to 50. At the end of the surgery, anesthetics were stopped. Approximately 2 mg/kg sugammadex was infused to reverse neuromuscular blockade. Endotracheal extubation was performed when the patients opened their eyes spontaneously or by verbal commands, and adequate tidal volume and respiratory rate were recovered. When consciousness and respiration were stable, the patients were transferred to the postanesthetic care unit. On the 1st postoperative day, a modified Brice interview (Supplementary Table 2) (22) was performed to evaluate the occurrence of intraoperative awareness.

### Statistical analysis

The statistical analyses were performed using SPSS 25 (SPSS Inc., Chicago, USA). Data were collected and presented as mean ± SD or number (%). Up-and-down sequences were analyzed by the probit test, which enabled the ED50 and ED95 of the initial infusion rate and total dose of DEX in anesthesia induction with 95% confidence limits (CI). The ED50 of the initial infusion rate was also calculated as the mean of the independent crossovers of the initial infusion rate. HR, mean arterial pressure (MAP), and PSI changes were analyzed using one-way ANOVA.

## Results

**Figure 1:**
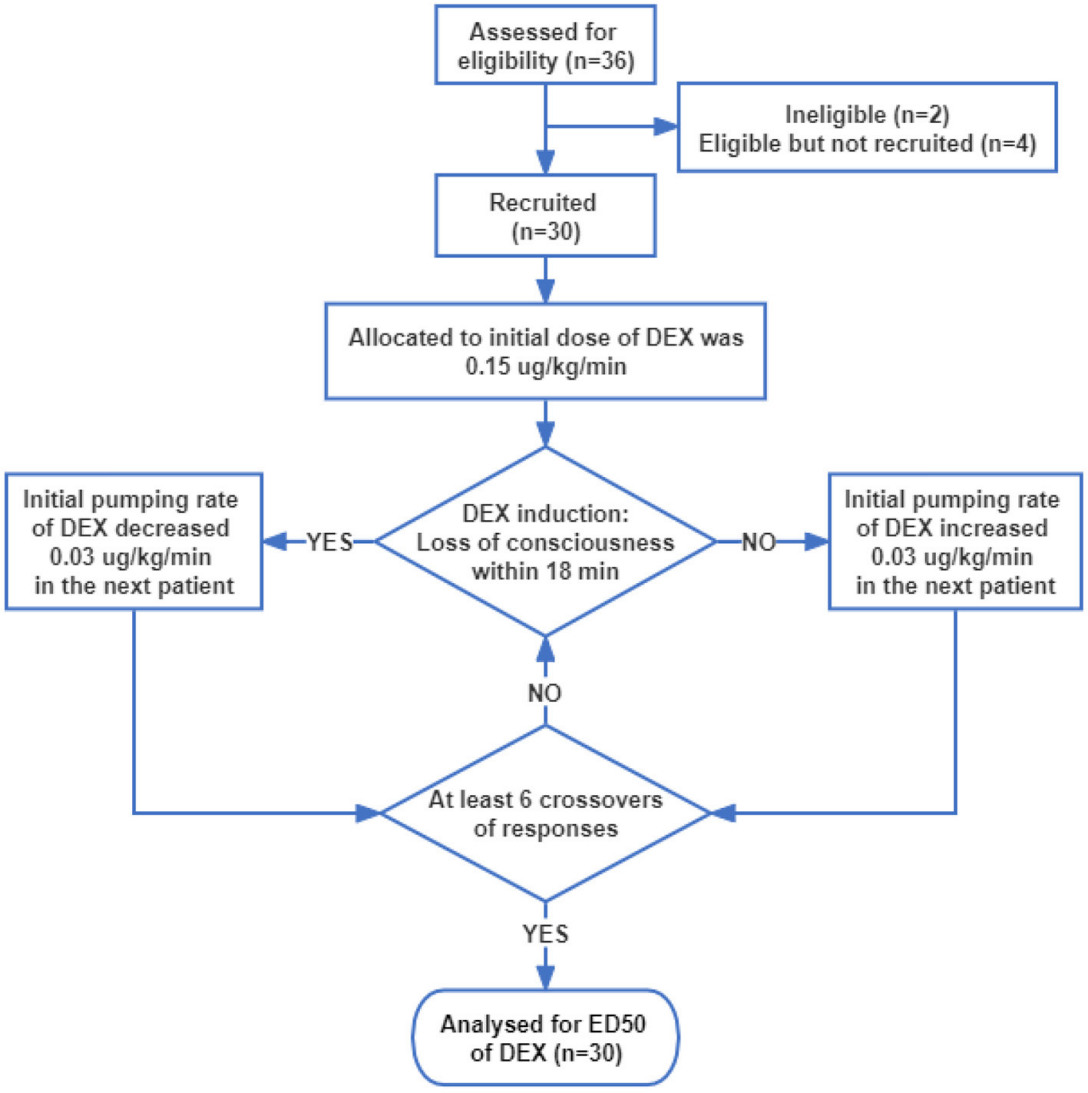
Flow chart for this Dixon's up-and-down sequential study.

The participants' flow during the study is shown in Figure 1. Thirty-six patients were assessed for eligibility, two patients did not meet the inclusion criteria and were excluded, and four patients were eligible but not recruited due to the postponement of surgery. Thus, 30 patients were recruited and completed the study. Patients' characteristics before surgery and baseline data are presented in Table 1, from which we can see the average age and BMI were 50.0 and 24.5, respectively. The average ALT, AST, creatinine, and urea nitrogen were within the normal range. All patients had MMSE scores >24, indicating that the preoperative cognitive function is intact. For preoperative comorbidities, 10 patients had a history of hypertension, three had diabetes, and one had obstructive sleep apnea syndrome (OSAS).

**Table 1:** Subject characteristics.

**Sex (Male/ Female)**	**15 / 15**
Age (year)	50.0 ± 7.8
Weight (kg)	70.4 ± 11.7
Height (cm)	169.0 ± 8.4
BMI (kg/m^2^)	24.5 ± 2.6
Temperature (^o^C)	36.3 ± 0.2
ALT (U/L)	22.0 ± 14.1
AST (U/L)	24.0 ± 14.0
Creatinine (μmol/L)	70.4 ± 19.5
Urea nitrogen (mmol/L)	5.1 ± 1.2
MMSE	29.0 ± 1.0
Smoking (%)	4 (13.3%)
Drinking (%)	5 (16.7%)
ASA grade (I/ II)	19 / 11
**Comorbidities**	
Hypertension	10 (33.3%)
Diabetes	3 (10.0%)
OSAS	1 (3.3%)
**Types of surgery**	
Gastric surgery	6 (20.0%)
Colorectal surgery	17 (56.7%)
Gynecologic surgery	7 (23.3%)

Data are presented as mean ± SD or number (%).

**Figure 2:**
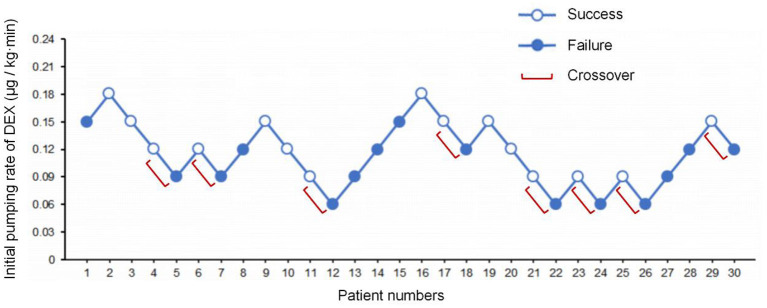
The sequence of initial infusion rate of DEX administered and subsequent response (loss of consciousness) within 18 min. Success – open circle; failure – filled circle.

The state of “loss of consciousness” was achieved within 18 min in 15 patients and was achieved more than 18 min in another 15 patients. The average induction time was 18.3 min. The consecutive results of the initial infusion rate are shown in Figure 2. Probit regression was used to obtain the ED50 and ED95 of DEX initial infusion rate for the first 9 min as 0.115 μg/kg/min (95% CI: 0.070–0.160 μg/kg/min) and 0.200 μg/kg/min (95% CI: 0.157–0.625 μg/kg/min), respectively. In total, eight crossover pairs appeared in this study, and the mean of the eight crossover pairs of initial infusion rate was 0.111 μg/kg/min, which is consistent with the result obtained by the probit regression. The dose of remifentanil to achieve the state of “loss of consciousness” was 1.83 ± 0.52 μg/kg.

**Figure 3:**
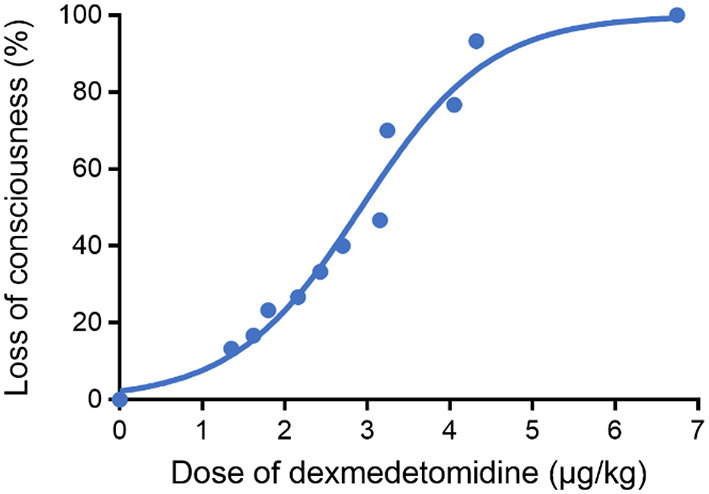
Dose–response curve for DEX plotted using probit analysis. The ED50 and ED95 of DEX to achieve the state of “loss of consciousness” were 2.899 (95% CI: 2.703–3.115 μg/kg) and 5.001 μg/kg (95% CI: 4.544–5.700 μg/kg), respectively.

As shown in Figure 3, a sigmoid dose–response curve was plotted by probit analysis with the number of responders at each dose. The estimated ED50 of DEX dose to achieve the state of “loss of consciousness” (OAA/S = 1) was 2.899 μg/kg (95% CI: 2.703–3.115 μg/kg) and the ED95 was 5.001 μg/kg (95% CI: 4.544–5.700 μg/kg). Table 2 shows the calculated doses of DEX at each probability of the state of “loss of consciousness”. The effect of DEX was dose-dependent, and the incidence of the response of “loss of consciousness” increased with the increasing dose. Furthermore, the influences of gender, ASA classification, and age on the effective dose of DEX were analyzed. The results indicated that there was no significant difference in the effective dose between male (3.26 ± 1.48 μg/kg) and female patients (3.08 ± 1.34 μg/kg, *p* = 0.729), between patients of ASA I (3.17 ± 1.58 μg/kg) and patients of ASA II (3.18 ± 1.04 μg/kg, *p* = 0.980), or between patients aged ≤ 50 years (3.38 ± 0.41 μg/kg) and patients aged > 50 years (3.04 ± 0.33 μg/kg, *p* = 0.511).

**Table 2:** Calculated probabilities of loss of consciousness.

**Probability**	**DEX dose (μg/kg)**	**95% CI**
0.05	0.841	0.309–1.204
0.10	1.295	0.871–1.593
0.20	1.846	2.539–2.077
0.30	2.242	2.002–2.443
0.40	2.582	2.377–2.778
0.50	2.899	2.703–3.115
0.60	3.215	3.008–3.473
0.70	3.555	3.317–3.873
0.80	3.951	3.666–4.354
0.90	4.502	4.138–5.033
0.95	4.956	4.523–5.599

**Figure 4:**
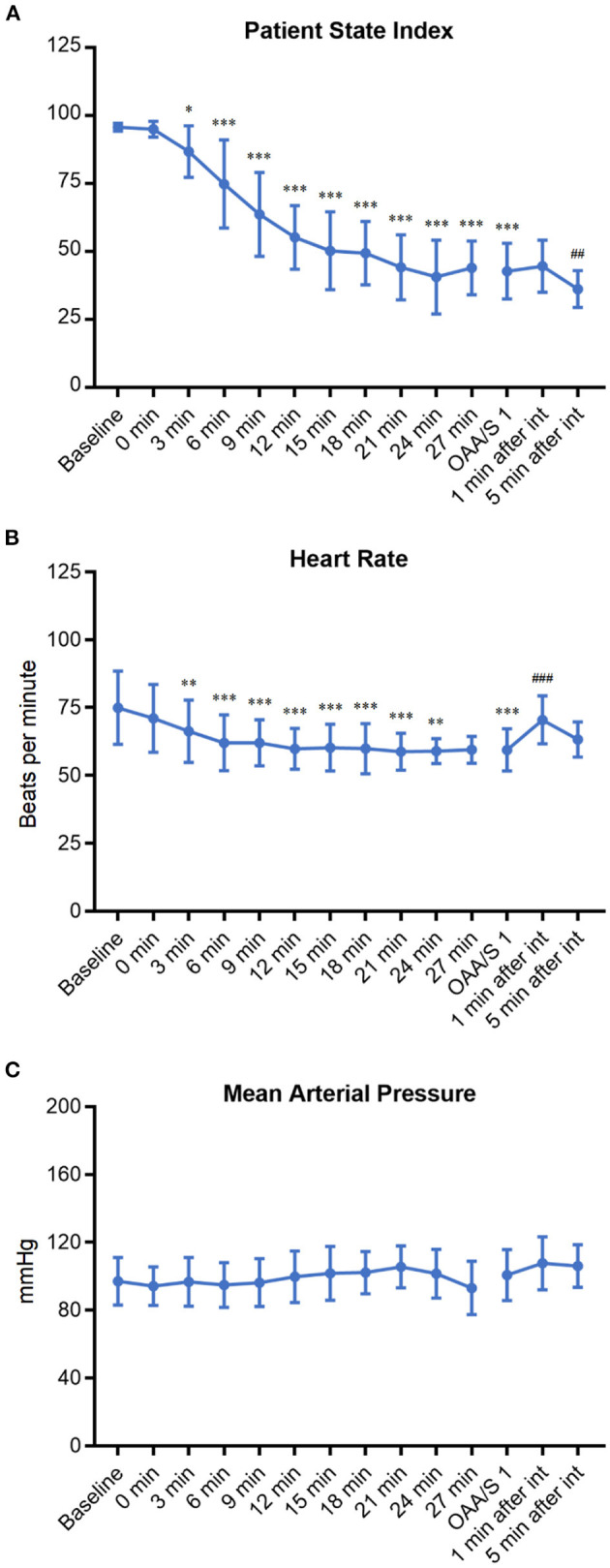
The change trends of PSI **(A)**, HR **(B)**, and MAP **(C)** of patients during DEX induction. *p < 0.05, **p < 0.01, and ***p < 0.001 compared with baseline; ^##^*p* < 0.01 and ^###^*p* < 0.001 compared with OAA/S 1 (before intubation).

To explore the situations and complications that may occur during DEX induction, the patient's characteristics in anesthesia induction were recorded and analyzed (Table 3 and Figure 4). The PSI scores at 3 min to 27 min after DEX pumping infusion decreased significantly compared with the baseline level (*p* < 0.05 or *p* < 0001). The average PSI on the loss of consciousness was 42.8 (< 50), which decreased significantly compared with the baseline level (*p* < 0.001). The PSI score at 5 min, but not 1 min, after intubation, decreased significantly compared with before intubation (*p* < 0.01, Figure 4A). The HR at 3 to 24 min after DEX pumping infusion decreased significantly compared with the baseline level (OAA/S 1, *p* < 0.01 or *p* < 0001). The average minimum and maximum HR during induction were 55.3 bpm and 78.2 bpm, respectively, and no bradycardia (HR < 45 bpm) occurred during the induction. The HR at 1 min after intubation increased significantly compared with before intubation (*p* < 0.001, Figure 4B). There were no significant variations in the average MAP of all patients during induction (Figure 4C), and the average minimum and maximum MAP was 87.6 mmHg and 107.4 mmHg, respectively. Three patients developed SBP > 180 mmHg during induction; they all had a history of hypertension, and their BP increases were <20% compared with the baseline. Approximately 100 μg of nitroglycerin was given and their BP returned to the normal range. No other vasoactive drugs were used during induction. Then, 5 L/min oxygen was provided with face masks during induction. Nine patients experienced SpO_2_ < 95% when they lost consciousness and the cause was considered glossoptosis. One patient had a history of OSAS, and his SpO_2_ decreased before he lost consciousness. All SpO_2_ returned to 100% after their lower jaws were lifted, and assisted ventilation was not necessary. A modified Brice interview was used on the 1st postoperative day to evaluate the occurrence of intraoperative awareness during anesthesia induction. The results indicated that the last thing to remember before induction was call name or facemask pre-oxygenation, and the first thing to remember after emergence was call name or in the ward in these patients. No intraoperative consciousness or dreaming was reported.

**Table 3:** DEX induction characteristics.

**Induction time (min)**	**18.3 ± 5.2**
PSI on loss of consciousness	42.8 ± 10.1
Minimum HR during induction (bpm)	55.3 ± 6.7 0
Bradycardia (HR < 45 bpm)	(0.0%)
Maximum HR during induction (bpm)	78.2 ± 11.5
Minimum MAP during induction (mmHg)	87.6 ± 10.8
Maximum MAP during induction (mmHg)	107.4 ± 13.5 3
Hypertension (SBP > 180 mmHg)	(10.0%)
SpO_2_ < 95% on loss of consciousness	10 (33.3%)
**The last thing to remember before induction**	
Call his/her name	15 (50.0%)
Facemask pre-oxygenation	15 (50.0%)
**The first thing to remember after emergence**	
Call his/her name in the operating room	18 (60.0%)
In the ward	12 (40.0%)
Conscious or dreaming during induction	0 (0.0%)
Adverse reactions during induction	0 (0.0%)

Data are presented as mean ± SD or number (%).

**Figure 5:**
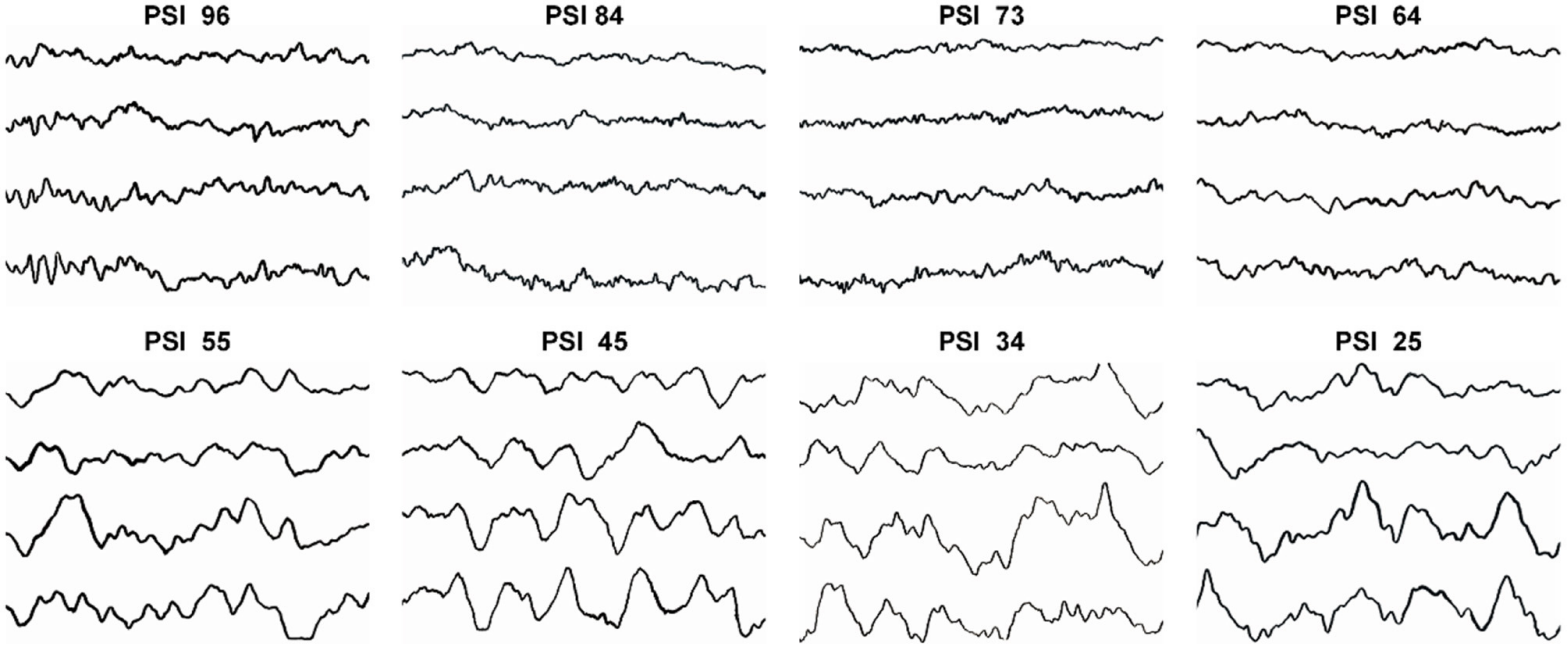
The typical patterns and the characteristic changes of EEG when PSI scores were 96, 84, 73, 64, 55, 45, 34, and 25 during DEX induction.

Figure 5 shows the typical patterns and the characteristic changes of EEG in the frontal and pre-frontal cortices of the brain during DEX induction. During the initial stage of DEX induction, when the depth of sedation was mild and PSI scores were high (96, 84, and 73), the typical EEG was mainly β and α waves. When the depth of sedation achieved moderate (PSI 64 and 55), β and α waves decreased, and θ wave increased gradually. When the depth of sedation further achieved the level of surgical anesthesia (PSI 45, 34, and 25), the EEG changed to θ wave and slow δ oscillations, which were the symbol of NREM sleep stage N3. The results indicated that the EEG changes during DEX-led anesthesia induction approximated the physiological sleep process.

## Discussion

This study indicated that continuous infusion of combined DEX and remifentanil could be an effective strategy for anesthesia induction. The ED50 and ED95 initial infusion rates of DEX were 0.115 and 0.200 μg/kg/min, respectively, for the first 9 min, the rate of DEX was adjusted two times and three times of initial infusion rate, respectively, for the second and third 9 min. The mean time to achieve the state of “loss of consciousness” was 18.3 min. The ED50 and ED95 of DEX to achieve the state of “loss of consciousness” in 18 min were 2.899 and 5.001 μg/kg, respectively. The hemodynamics including BP and HR were relatively stable among the patients, three patients (with a history and baseline hypertension) developed SBP > 180 mmHg during induction, and no patient developed bradycardia (HR < 45 bpm). Respiratory function remained intact throughout the process. Glosspotosis occurred in few patients and their SpO_2_ returned to 100% after the jaws were lifted without assisted ventilation. No intraoperative awareness or other adverse reactions during the induction period were reported. The mean PSI on the loss of consciousness was 42.8. EEG showed that during DEX induction, α and β power decreased, while θ and δ power increased in the frontal and pre-frontal cortices of the brain, which was similar to the physiological sleep process.

The purpose of this study was to establish an effective strategy with DEX to achieve the state of “loss of consciousness” in anesthesia induction. As a previous study reported, the administration of a single bolus DEX has a 6.5 ± 3.4 min distribution half-life, and its anesthesia onset time is even longer (23). Loading doses of 0.5–1.0 μg/kg DEX, followed by an infusion at a rate of 0.2–0.7 μg/kg/h, could provide effective sedation (Ramsay sedation score ≥ 3) and be well-tolerated (24). Our preliminary experiment indicated that shortening the induction time to <10 min may trigger bradycardia (HR < 45 bpm) and hypertension (SBP > 180 mmHg). Therefore, the threshold of Dixon's up-and-down method was extended to 18 min. The initial infusion rate was set at a relatively low rate for the first 9 min and increased gradually for the second and third 9 min to control hemodynamic changes during induction. With this strategy, the dose of DEX was greater than the amount used in previous studies or clinical practices but no patient experienced bradycardia.

The main reason for using the remifentanil combination was that induction with DEX alone required a higher dose and prolonged time, which raised the occurrence of hypertension in the preliminary experiment (Up to 30%). This combination let alone with a lower DEX dose and relatively shorter induction duration. The opioids also help to improve the patient's tolerance to tracheal intubation, and a study showed that 0.025–0.15 μg/kg/min remifentanil could be safely used for birth pain relief, and no patients experienced SpO_2_ ≤ 95% without oxygen supplementation (25). Thus, combined with a low-rate infusion of remifentanil could be an effective strategy to control the DEX dose during induction without obvious respiratory depression. Remifentanil could also decrease the occurrence of hypertension, and in this study, only three patients with a history of hypertension developed SBP > 180 mmHg during induction, and their BP fluctuations were <20% compared with the baseline level.

Dexmedetomidine induction took a longer time, the SpO_2_ was stable until the state of “loss of consciousness” was achieved and glossoptosis occurred. The SpO_2_ returned to 100% after the jaw was raised without assisted ventilation, which indicated that DEX had a respiratory-preserving property. Previous studies indicated that infusion of 2 ug/kg DEX did not decrease the respiratory rate in volunteers, although the tidal volume was slightly reduced (26). The effect of DEX on breathing is much less pronounced than opioids and other intravenous sedation drugs, and the degree of breathing is similar to that of deep sleep (27). Because of this feature, DEX-induced cooperative sedation with minimal respiratory depression provides safe and acceptable conditions during neurosurgical procedures in awake patients and awake fiberoptic intubation (28).

Sleep is a rapidly reversible state of reduced responsiveness and metabolism of the brain, which can be broadly segmented into rapid eye movement (REM) sleep and non-REM (NREM) sleep. NREM sleep comprises the majority of the total sleep time in adults. It could be further divided into three sub-stages: stage N1, stage N2, and stage N3. Stage N1 sleep is the transition stage from wakefulness to sleep, which is characterized by decreased α rhythm and low amplitude EEG pattern in the theta range. During stage N2 sleep, spindle powers and K-complexes appear. Stage N3 sleep, also referred to as “deep sleep” or “slow wave sleep”, is characterized by low frequency but high amplitude δ EEG waves (29). General anesthesia-induced unconsciousness has been compared with sleep (30). The anesthetic state and sleep have similarities and differences in EEG dynamics (31). In the propofol-induced loss of consciousness process, an increase in low-frequency power and the appearance of coherent frontal alpha oscillations were observed, but with shorter periods of neuronal firing and longer periods of silence than sleep slow waves (32), which suggest a more profound degree of cortical impairment than natural sleep. The present results showed that as anesthesia depth increased during DEX induction, α and β power decreased, while θ and δ power increased in the frontal and pre-frontal cortices of the brain. Previous results showed that DEX-induced spectral power of moderate sedation was larger at 0.75–6.5 Hz and 12.5–15.5 Hz and smaller at 8.5–11 Hz and 17.5–40 Hz (33). DEX promoted N3 sleep in a dose-dependent manner and did not impair performance on the psychomotor vigilance test (5). These data indicated that DEX anesthesia was more similar to NREM sleep stage N3 than propofol anesthesia, and EEG pattern scores could be used to evaluate the depth of DEX anesthesia.

About 25% of patients aged over 60 years experienced POCD within 7 days after major noncardiac surgery (34). Dose-related neurotoxic effects of inhaled anesthetics and propofol have been reported in animal experiments (35–37). Studies suggested that systemic inflammation response was a leading trigger (38). DEX could suppress stress responses and accompanying neuroinflammation (39), and reduce the emergence of delirium in both adults and children (40). DEX could also induce EEG changes similar to NREM sleep stage N3, but propofol could produce burst suppression, a pattern of intermittent bursts of activity punctuated by electrical silence, which is not observed during natural sleep. Studies suggested that burst suppression or low BIS values were associated with postoperative delirium (41, 42). Therefore, DEX induction and even maintenance may lead to less neurotoxicity and improved postoperative recovery, especially in elderly or recognition-impaired patients.

There are several limitations to this study and induction strategy. First, in the preliminary experiment, we found that induction with DEX alone led to hypertension (SBP > 180 mmHg) in 30% of patients, then the induction was performed with continuous infusion of both DEX and remifentanil. Thus, the results were not the effective dose for the sole application of DEX. Second, to avoid bradycardia, the induction time was extended. Although the induction process and hemodynamics were stable, 18 min induction time was relatively long in clinical practice. Third, this study was launched in relatively healthy patients (ASA I or II, baseline HR > 50 bpm), and these patients were not receiving any other common vasoactive agents (such as beta-blockers, calcium channel blockers, etc.) that might potentiate the hemodynamic effect of DEX. This alleviated the generalization of the results to common practice. Additional studies with different DEX loading doses in different disease settings are necessary. Fourth, this study is an investigation of the clinical performance of DEX-led anesthesia induction, and PD/PK models detection and calculation would be necessary for future studies. Finally, the state of “loss of consciousness” and depth of anesthesia were mainly measured with a subjective method ‘OAA/S', and PSI was only used as an indicator. Although no awareness was reported, the depth of anesthesia still needs further investigations with more monitors, including BIS and EEG.

## Conclusion

This study indicated that continuous infusion of combined DEX and remifentanil could be an effective strategy for anesthesia induction. The ED50 and ED95 of DEX to achieve the state of “loss of consciousness” were 2.899 and 5.001 μg/kg, respectively. The initial infusion rate of DEX was 0.115 μg/kg/min and the mean induction time was 18.3 min. The hemodynamics including BP and HR were stable among the patients, and the mean PSI on the loss of consciousness was 42.8. EEG showed that during DEX induction, α and β power decreased, while θ and δ power increased in the frontal and pre-frontal cortices of the brain, which was similar to the spontaneous sleep process. However, the application of DEX in general anesthesia still needs further investigation, as well as the mechanism and relationship between general anesthesia and sleep.

## Data availability statement

The original contributions presented in the study are included in the article/Supplementary material, further inquiries can be directed to the corresponding authors.

## Ethics statement

The studies involving human participants were reviewed and approved by the Ethics Committee of National Cancer/Cancer Hospital, Chinese Academy of Medical Sciences and Peking Union Medical College (Approval No. 21/024-2695). The patients/participants provided their written informed consent to participate in this study.

## Author contributions

BM contributed to the experiment design and execution and manuscript revision. WX and HL contributed to the experiment and data analysis and manuscript draft and revision. ZS and YZ contributed to data analysis and manuscript revision. XW performed the statistical analysis. BZ, JY, and NT contributed to the experiment execution and manuscript revision. NL contributed to EEG and data analysis. DZ and ZZ contributed to the experiment execution. HZ contributed to the project design and supervision and manuscript revision. CN designed the project, performed the experiments and data collection, and drafted and revised the manuscript. All authors contributed to the article and approved the submitted version.

## References

[1] Mantz J Josserand J Hamada S. Dexmedetomidine: new insights. Eur J Anaesthesiol. (2011) 28:3–6. 10.1097/EJA.0b013e32833e266d 20881501

[2] Jorm CM Stamford JA. Actions of the hypnotic anaesthetic, dexmedetomidine, on noradrenaline release and cell firing in rat locus coeruleus slices. Br J Anaesth. (1993) 71:447–9. 10.1093/bja/71.3.447 8104450

[3] Nelson LE Lu J Guo T Saper CB Franks NP Maze M. The alpha2-adrenoceptor agonist dexmedetomidine converges on an endogenous sleep-promoting pathway to exert its sedative effects. Anesthesiology. (2003) 98:428–36. 10.1097/00000542-200302000-00024 12552203

[4] Brown EN Purdon PL Van Dort CJ. General anesthesia and altered states of arousal: a systems neuroscience analysis. Annu Rev Neurosci. (2011) 34:601–28. 10.1146/annurev-neuro-060909-153200 21513454 PMC3390788

[5] Akeju O Hobbs LE Gao L Burns SM Pavone KJ Plummer GS. Dexmedetomidine promotes biomimetic non-rapid eye movement stage 3 sleep in humans: a pilot study. Clin Neurophysiol. (2018) 129:69–78. 10.1016/j.clinph.2017.10.005 29154132 PMC5743618

[6] Carr ZJ Cios TJ Potter KF Swick JT. Does dexmedetomidine ameliorate postoperative cognitive dysfunction? A brief review of the recent literature. Curr Neurol Neurosci Rep. (2018) 18:64. 10.1007/s11910-018-0873-z 30083844

[7] Wesselink EM Kappen TH Torn HM Slooter AJC van Klei WA. Intraoperative hypotension and the risk of postoperative adverse outcomes: a systematic review. Br J Anaesth. (2018) 121:706–21. 10.1016/j.bja.2018.04.036 30236233

[8] Schonberger RB Dai F Michel G Vaughn MT Burg MM Mathis M. Association of propofol induction dose and severe pre-incision hypotension among surgical patients over age 65. J Clin Anesth. (2022) 80:110846. 10.1016/j.jclinane.2022.110846 35489305 PMC11150018

[9] Lin N Vutskits L Bebawy JF Gelb AW. Perspectives on dexmedetomidine use for neurosurgical patients. J Neurosurg Anesthesiol. (2019) 31:366–77. 10.1097/ANA.0000000000000554 30363004

[10] Patel M Onwochei DN Desai N. Influence of perioperative dexmedetomidine on the incidence of postoperative delirium in adult patients undergoing cardiac surgery. Br J Anaesth. (2022) 129:67–83. 10.1016/j.bja.2021.11.041 35279278

[11] Zhang Z Ferretti V Güntan I Moro A Steinberg EA Ye Z. Neuronal ensembles sufficient for recovery sleep and the sedative actions of α2 adrenergic agonists. Nat Neurosci. (2015) 18:553–61. 10.1038/nn.3957 25706476 PMC4836567

[12] Ramsay MA Luterman DL. Dexmedetomidine as a total intravenous anesthetic agent. Anesthesiology. (2004) 101:787–90. 10.1097/00000542-200409000-00028 15329604

[13] Yamanaka D Kawano T Nishigaki A Aoyama B Tateiwa H Shigematsu-Locatelli M. Preventive effects of dexmedetomidine on the development of cognitive dysfunction following systemic inflammation in aged rats. J Anesth. (2017) 31:25–35. 10.1007/s00540-016-2264-4 27738803

[14] Yin D Zhou S Xu X Gao W Li F Ma Y. Dexmedetomidine attenuated early brain injury in rats with subarachnoid haemorrhage by suppressing the inflammatory response: The TLR4/NF-κB pathway and the NLRP3 inflammasome may be involved in the mechanism. Brain Res. (2018) 1698:1–10. 10.1016/j.brainres.2018.05.040 29842860

[15] Tasbihgou SR Barends CRM Absalom AR. The role of dexmedetomidine in neurosurgery. Best Prac Res Clin Anaesthesiol. (2021) 35:221–9. 10.1016/j.bpa.2020.10.002 34030806

[16] Kaya FN Yavascaoglu B Turker G Yildirim A Gurbet A Mogol EB. Intravenous dexmedetomidine, but not midazolam, prolongs bupivacaine spinal anesthesia. J Can Anesthesie. (2010) 57:39–45. 10.1007/s12630-009-9231-6 20039221

[17] Dutta A Sethi N Sood J Panday BC Gupta M Choudhary P. The effect of dexmedetomidine on propofol requirements during anesthesia administered by bispectral index-guided closed-loop anesthesia delivery system: a randomized controlled study. Anesth Analg. (2019) 129:84–91. 10.1213/ANE.0000000000003470 29787410

[18] Ebert TJ Hall JE Barney JA Uhrich TD Colinco MD. The effects of increasing plasma concentrations of dexmedetomidine in humans. Anesthesiology. (2000) 93:382–94. 10.1097/00000542-200008000-00016 10910487

[19] Zhang T Deng Y He P He Z Wang X. Effects of mild hypoalbuminemia on the pharmacokinetics and pharmacodynamics of dexmedetomidine in patients after major abdominal or thoracic surgery. J Clin Anesth. (2015) 27:632–7. 10.1016/j.jclinane.2015.06.002 26277872

[20] Castillo RL Ibacache M Cortínez I Carrasco-Pozo C Farías JG Carrasco RA. Dexmedetomidine improves cardiovascular and ventilatory outcomes in critically ill patients: basic and clinical approaches. Front Pharmacol. (2019) 10:1641. 10.3389/fphar.2019.01641 32184718 PMC7058802

[21] Talke P Anderson BJ. Pharmacokinetics and pharmacodynamics of dexmedetomidine-induced vasoconstriction in healthy volunteers. Br J Clin Pharmacol. (2018) 84:1364–72. 10.1111/bcp.13571 29495085 PMC5980451

[22] Mashour GA Kent C Picton P Ramachandran SK Tremper KK Turner CR. Assessment of intraoperative awareness with explicit recall: a comparison of 2 methods. Anesth Analg. (2013) 116:889–91. 10.1213/ANE.0b013e318281e9ad 23460567

[23] Anttila M Penttilä J Helminen A Vuorilehto L Scheinin H. Bioavailability of dexmedetomidine after extravascular doses in healthy subjects. Br J Clin Pharmacol. (2003) 56:691–3. 10.1046/j.1365-2125.2003.01944.x 14616431 PMC1884292

[24] Ko KH Jun IJ Lee S Lim Y Yoo B Kim KM. Effective dose of dexmedetomidine to induce adequate sedation in elderly patients under spinal anesthesia. Korean J Anesthesiol. (2015) 68:575–80. 10.4097/kjae.2015.68.6.575 26634081 PMC4667143

[25] D'Onofrio P Novelli AM Mecacci F Scarselli G. The efficacy and safety of continuous intravenous administration of remifentanil for birth pain relief: an open study of 205 parturients. Anesth Analg. (2009) 109:1922–4. 10.1213/ane.0b013e3181acc6fc 19641051

[26] Belleville JP Ward DS Bloor BC Maze M. Effects of intravenous dexmedetomidine in humans. I Sedation, ventilation, and metabolic rate. Anesthesiology. (1992) 77:1125–33. 10.1097/00000542-199212000-00013 1361310

[27] Venn RM Hell J Grounds RM. Respiratory effects of dexmedetomidine in the surgical patient requiring intensive care. Critical Care. (2000) 4:302–8. 10.1186/cc712 11056756 PMC29047

[28] S. Lee. Dexmedetomidine: present and future directions. Korean J Anesthesiol. (2019) 72:323–30. 10.4097/kja.19259 31220910 PMC6676029

[29] Iber C Ancoli-Israel S Chesson AL Quan SF. The AASM manual for the scoring of sleep and associated events. rules, terminology and technical specifications, Darien, Illinois. Am Acad Sleep Med. (2012) 176:2012.

[30] Franks NP Zecharia AY. Sleep and general anesthesia. Can J Anaesth. (2011) 58:139–48. 10.1007/s12630-010-9420-3 21170623

[31] Brown EN Lydic R Schiff ND. General anesthesia, sleep, and coma. N Engl J Med. (2010) 363:2638–50. 10.1056/NEJMra0808281 21190458 PMC3162622

[32] Purdon PL Pierce ET Mukamel EA Prerau MJ Walsh JL Wong KF. Electroencephalogram signatures of loss and recovery of consciousness from propofol. Proc Natl Acad Sci U S A. (2013) 110:E1142–51. 10.1073/pnas.1221180110 23487781 PMC3607036

[33] Xi C Sun S Pan C Ji F Cui X Li T. Different effects of propofol and dexmedetomidine sedation on electroencephalogram patterns: wakefulness, moderate sedation, deep sedation and recovery. PLoS ONE. (2018) 13:e0199120. 10.1371/journal.pone.0199120 29920532 PMC6007908

[34] Moller JT Cluitmans P Rasmussen LS Houx P Rasmussen H Canet J. Long-term postoperative cognitive dysfunction in the elderly ISPOCD1 study. ISPOCD investigators international study of post-operative cognitive dysfunction. Lancet. (1998) 351:857–61. 10.1016/S0140-6736(97)07382-0 9525362

[35] Cattaneo A Cattane N Galluzzi S Provasi S Lopizzo N Festari C. Association of brain amyloidosis with pro-inflammatory gut bacterial taxa and peripheral inflammation markers in cognitively impaired elderly. Neurobiol Aging. (2017) 49:60–8. 10.1016/j.neurobiolaging.2016.08.019 27776263

[36] Ni C Qian M Geng J Qu Y Tian Y Yang N. DNA Methylation manipulation of memory genes is involved in sevoflurane induced cognitive impairments in aged rats. Front Aging Neurosci. (2020) 12:211. 10.3389/fnagi.2020.00211 33013350 PMC7461785

[37] Zhou H Xie Z Brambrink AM Yang G. Behavioural impairments after exposure of neonatal mice to propofol are accompanied by reductions in neuronal activity in cortical circuitry. Br J Anaesth. (2021) 126:1141–56. 10.1016/j.bja.2021.01.017 33641936 PMC8216302

[38] Subramaniyan S Terrando N. Neuroinflammation and Perioperative Neurocognitive Disorders. Anesth Analg. (2019) 128:781–8. 10.1213/ANE.0000000000004053 30883423 PMC6437083

[39] Wang K Wu M Xu J Wu C Zhang B Wang G. Effects of dexmedetomidine on perioperative stress, inflammation, and immune function: systematic review and meta-analysis. Br J Anaesth. (2019) 123:777–94. 10.1016/j.bja.2019.07.027 31668347

[40] Duan X Coburn M Rossaint R Sanders RD Waesberghe JV Kowark A. Efficacy of perioperative dexmedetomidine on postoperative delirium: systematic review and meta-analysis with trial sequential analysis of randomised controlled trials. Br J Anaesth. (2018) 121:384–97. 10.1016/j.bja.2018.04.046 30032877

[41] Evered LA Chan MTV Han R Chu MHM Cheng BP Scott DA. Anaesthetic depth and delirium after major surgery: a randomised clinical trial. Br J Anaesth. (2021) 127:704–12. 10.1016/j.bja.2021.07.021 34465469 PMC8579421

[42] Pedemonte JC Plummer GS Chamadia S Locascio JJ Hahm E Ethridge B. Electroencephalogram burst-suppression during cardiopulmonary bypass in elderly patients mediates postoperative delirium. Anesthesiology. (2020) 133:280–92. 10.1097/ALN.0000000000003328 32349072 PMC7365754

