# Supplementary material for: Determination of the effective dose of dexmedetomidine to achieve loss of consciousness during anesthesia induction
Source: Front Med (Lausanne). 2023 Apr 20;10:1158085. doi: 10.3389/fmed.2023.1158085 (PMC10159180; doi:10.3389/fmed.2023.1158085)
Supplement: Supplementary file 1 [file Table_1.DOCX]

Supplementary table1 Observer’s Assessment of Alertness/Sedation (OAA/S) scale

| Score | Responsiveness | Speech | Facial expression | Eyes |
| --- | --- | --- | --- | --- |
| 1 | Does not respond to mild prodding or shaking |  |  |  |
| 2 | Responds only after mild prodding or shaking | Few recognized words |  |  |
| 3 | Responds only after the name is spoken loudly and/or repeatedly | Slurring or prominent slowing | Marked relaxation  (slack jaw) | Glazed and marked ptosis |
| 4 | Lethargic response to name spoken in a normal tone | Mild slowing or thickening | Mild relaxation | Glazed or mild ptosis |
| 5 | Responds readily to name spoken in normal tone | Normal | Normal | Clear, no ptosis |

The final score is the sum of the Responsiveness, Speech, Facial expression, and Eyes component scores. Thus, a ‘wide awake’ score=5 and a ‘deeply sedated’ score=1.

Supplementary table 2 Modified Brice Interview 24

| 1. What was the last thing you remember before anesthesia?  2. What is the first thing you remember after waking up?  3. Do you remember anything between going under anesthesia and waking up?  4. Did you dream during your procedure? |
| --- |
| 5. What was the worst thing about your operation? |
